# Probing Evolutionary Biography of MicroRNAs and Associated Factors

**DOI:** 10.2174/138920212799860634

**Published:** 2012-04

**Authors:** Bibekanand Mallick, Zhumur Ghosh

**Affiliations:** 1 Wadsworth Center, New York State Department of Health, Albany, NY, USA; 2 Bioinformatics Centre, Bose Institute, Kolkata, India

**Keywords:** miRNA evolution, biogenesis, target specificity, seed shifting, arm switching, duplication, noncoding RNA.

## Abstract

Intergenic DNA, often described as “playground of evolution”, harbors a plethora of cis and trans regulatory elements in the form of non-coding RNAs (ncRNAs). The evolution of the silencing mechanism mediated by microRNAs (miRNAs), an important class of ncRNA, involves the proliferation of miRNA biogenesis and effector proteins, continuing innovation of novel families by the diversification of established families and spawning additional paralogous family members. Such evolving miRNA pathways for spatiotemporal regulation of the transcriptome have shaped the evolution of eukaryotic genomes and contributed to the complexity of multicellular organisms. Here, we focus on the emergence of new target specificity of the miRNAs along with the proliferation of core biogenesis and effector modules and show how this has contributed to generate diverse miRNA regulatory pathways.

## INTRODUCTION

MicroRNAs (miRNAs) are an abundant class of small, ~22-nt-long endogenous, single-stranded non-coding RNAs (ncRNAs) that govern the post-transcriptional gene silencing through deadenylation, translational repression, and decay of their target messenger RNAs (mRNAs) [1-3]. They play critical roles in diverse biological processes in plants and animals, including developmental timing, embryogenesis, cell differentiation, cell proliferation, organogenesis, apoptosis, and tumorigenesis [3-7]. The deregulation and aberrant expression of miRNAs are also implicated in the risk of several diseases, including cancer [8]. Often evolutionary conserved, these tiny regulatory RNAs suppress the translation and/or promote the degradation of target mRNAs through incomplete complementary binding, primarily to 3´untranslated region (UTR) and sometimes confer repression (although significantly less) by binding to coding sequence (CDS) and 5´UTR [9]. The complementary base pairing of the miRNA guides RISC (RNA-induced silencing complex) of Argonaute:miRNA complex to target mRNAs, which are then degraded, destabilized or translationally inhibited by the Ago proteins [10-12]. Each miRNA is able to target several hundreds of different mRNAs. On the other hand, a mRNA may be simultaneously targeted by multiple miRNAs. More than 30% of human genes appear to be under selective pressure of being targeted by miRNAs [13]. These facts highlight the versatility and complexity of gene regulation by miRNAs, which is quite appealing as a scientific challenge to decipher and understand the biological systems more precisely through noology of miRNAs.

Lin-4, the first miRNA was discovered *in C. elegans* in 1993 by forward genetic approaches [14]. This was found to negatively regulate the expression of lin-14 mRNA through interaction with a complementary region in 3´UTR of lin-14. Although it was thought to be unique, discovery of let-7 in the same species after 7 years [15], followed by subsequent identification of several let-7 homologs in other groups of bilateral animals including mammals, brought a new renaissance that considered these miRNAs as a novel class of regulatory genes. Since then, miRNAs have been identified in plants, animals and viruses by the combination of experimental, computational and high-throughput sequencing approaches. This has resulted in the discovery of over 16,000 miRNA gene loci encoding over 19,000 distinct mature miRNAs in over 150 species [16] and they are now deposited in the central repository of miRNA, miRBase.

A significant fraction of the miRNA repertoire of any species is conserved with other species [17]. The target sites in 3´ UTR are also largely conserved across species [13]. This feature is one of the most important criteria for target discovery and has been included in most of the target identification algorithms [18-19]. miRNAs are continuously added to the metazoan genomes since geologic times, but are rarely known to undergo secondary loss because of intense negative selection [20-22]. miRNAs are involved in maintaining genic precision and considered to have some role in the development of biological robustness [23]. The vertebrates exhibit a diverse set of miRNAs, *Homo sapiens* tops the list as compared to bilaterian invertebrates [22]. These evolutionary features supported by continuous expansion of miRNA repertoire, especially in complex organisms, suggest that miRNAs might be excellent phylogenetic markers and could be useful in exploring the root of molecular basis of morphological complexity [24] and developmental canalization [23]. Canalization refers to the process of evolution of robustness that decreases inter-individual variability. Many evolutionary biologists have focused their research on evolutionary genomics of miRNAs and their targets in the last decade, however, many aspects of evolution still remain to be explored. In this article, we will address various evolutionary patterns/mechanisms in miRNAs, their targets and implications in the evolution of complex gene regulatory networks mediated by these micro molecules. 

## BIOGENESIS OF miRNAs

**Fig. (1):**
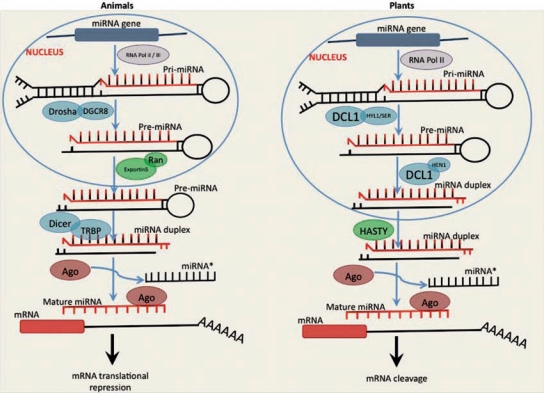
Schematic representation of miRNA biogenesis in animals and plants.

miRNAs are mainly encoded in either intergenic regions or within protein coding genes, usually within introns [25, 26]. The intergenic miRNA genes are transcribed into primary miRNA transcripts (pri-miRNA) by either RNA polymerase II or RNA polymerase III [27, 28] and are believed to be under the control of a specific promoter (Fig.**1**). In contrast, intronic miRNAs are co-transcribed with their host genes [25]. Many pri-miRNAs are polyadenylated and capped as observed in mRNAs, which indicate that the process is mediated by RNA Pol II. However, the largest human miRNA cluster, C19MC, is transcribed by RNA Pol III [28, 29]. Murid herpesvirus 4 (MuvHV-4) also generates pri-miRNAs through RNA Pol III transcription [28]. The subsequent steps in miRNA biogenesis in different groups of organisms and proteins/enzymes involved in the process, will be elaborated from evolutionary point of view in the next few sections. 

### Drosha and Accessory Proteins

In mammals, nuclear microprocessor complex formed by an RNase III enzyme called Drosha and its regulatory sub-unit DGCR8 (DiGeorge critical region) cleaves the pri-miRNA transcript to generate a hairpin precursor (pre-miRNA) in the nucleus [30]. The pri-miRNAs of metazoa contain a hairpin stem of ~33 base pairs (bps), two unpaired flanking sequence upstream and downstream of the hairpin and a terminal loop [31]. Drosha cleaves ~11 bps away from the single-stranded RNA/double-stranded RNA junction at the base of the hairpin. The hairpin stem and flanking sequences act as recognition elements for binding of DGCR8, the molecular ruler that determines the precision of cleavage site during Drosha processing [31]. In some cases, loops of pri-miRNAs also act as a site of binding for nuclear ribonucleoprotein A1 (hnRNP A1), which in turn, changes the conformation of hairpin to create a more favorable cleavage site for Drosha processing [32]. It is estimated that ~14% of the human pri-miRNA loops are conserved across different species. These conserved loops could have some evolutionary implications and further study may provide some clue on its significance in Drosha processing. Fig. (**2**) shows the phylogenetic tree of Drosha in vertebrates. The Drosha-mediated processing was initially thought to be a universal phenomenon; however, mirtrons, a recent class of miRNAs produced from intronic hairpins through a distinct pathway is found to deviate and bypass this process. This drosha-independent pathway is now reported in several organisms, such as flies, worms, and mammals [33].

Drosha as well as DGCR8 are conserved among vertebrates, including humans, mice, rats, chickens, and zebrafish [34, 35]. In plants, Drosha homologs are not observed, suggesting that the miRNA biogenesis pathway is distinct and different from animals (Fig. **1**). The pri-miRNAs transcribed from miRNA genes in plants are excised by dicer-like 1 (DCL1) in a step-wise manner, with the help of a dsRNA-binding protein Hyponastic leaves 1 (HYL1) and zinc-finger protein Serrate (SE) to generate a miRNA-miRNA* duplex [36]. HYL1 is required for a précise processing of pri-miRNAs in plants. However, the mechanism of action of HYL1 still remains elusive. This protein might be playing the role of DGCR8 for DCL1. DCL1 contains two double-stranded RNA-binding domains, whereas Drosha contains only one. This explains why the miRNA biogenesis machinery in plants and animals is significantly different (Fig.**1**). DCL1 performs a dual role of Drosha and Dicer as performed by these two together in animals. 

In plants, miRNA-miRNA* duplex is then exported into the cytoplasm by the HASTY (an Exportin-5 homolog) after methylation by methyltransferase HUA enhancer 1 (HEN1) [37]. In the cytoplasm, mature miRNA strand associates with Ago1 to form RISC [38]. In animals, the pre-miRNA produced in the nucleus through Drosha processing is exported into the cytoplasm by Exportin-5 and its Ran cofactor bound to GTP. Typically, pre-miRNAs are ~70-nt long, comprising of a stem of ~24-bp, a loop of variable size, and a 3′ overhang of ~2-nt [39]. The double-stranded stem and the 3´ overhangs of pre-miRNAs act as a structural motif to facilitate Exportin-5 binding and subsequent export into the cytoplasm. Once in the cytoplasm, pre-miRNAs undergo two different fates based on the degree of complementarity along the hairpin stem. In case of a high degree of complementarity, pre-miRNA undergoes cleavage of its passenger strand by Ago2, and generates a nicked hairpin, called ac-pre-miRNA (Ago2-cleaved pre-miRNA) [40]. The pre-miRNA or ac-pre-miRNA is then cleaved by another conserved RNase III, Dicer [41, 42] and a miRNA duplex is generated that is approximately 22-bp long with 2 nts protruding as overhangs at each 3´ end. This miRNA duplex could give rise to two different mature miRNAs, but only one strand of the duplex is usually loaded into an Argonaute family protein (AGO) to form the core of miRNA-induced silencing complexes (miRISCs). The other strand is degraded. The decision of incorporation of either strands depends on thermodynamic stability of the 5^/^ region of the miRNA in the duplex [43]. The mRNA targets to be silenced by miRISCs are selected through complementary base pairing interactions (partial or full) between the loaded miRNA and 3´ UTR of the target.

### Dicer and Accessory Proteins

Dicer and accessory proteins, as mentioned earlier, act on pre-miRNAs and complete the process of miRNA biogenesis. This process is not as simple as it appears to be because similar types of silencing RNAs (such as siRNA etc), other than miRNAs, compete with each other for processing by Dicer. Therefore, different organisms have evolutionarily acquired different approaches to recruit specific small ncRNAs. In animals, a single Dicer performs catalysis of the formation of multiple classes of small ncRNAs by switching the interactions with other accessory proteins. Dicer in mammals is aided by *trans*-activation responsive RNA-binding protein (TRBP) [44] for specialized processing. In worms, Dicer 1 (DCR1), a functional ortholog of the Drosophila DCR1 [41], requires DCR-1-interacting proteins and Argonaute-like 1 and 2 (ALG-1 and ALG-2) for miRNA processing [45, 46]. There are other cases where specificity of Dicer results from the existence of multiple members of Dicer-family proteins in a single process. Additionally, it has been reported that Dicer is not an obligatory protein for this purpose as it was thought earlier. Dicer-independent miRNA biogenesis is recently observed where the pre-miRNA load into Ago and is cleaved by the Ago2 catalytic center to generate an intermediate 3′ end, which is then further trimmed [47].

### Argonaute Protein Family

The Argonaute protein family is the key player of gene-silencing pathways. This protein family is characterized by the presence of three domains: PAZ (Piwi-Argonaute-Zwille), Mid and PIWI (P-element-induced wimpy testes) [48]. Argonaute proteins are highly conserved between species and can be classified into three paralogous groups: (a) AGO subfamily, which is often associated with the RNA duplexes processed by RNase III, (b) Piwi subfamily, which is involved in the transcriptional gene silencing of retrotransposons and other genetic elements in germ line cells, and (c) WAGO (Worm specific AGO) in worms [49]. AGO and Piwi proteins are widely found in all the three major domains of life, i.e. bacteria, archaea and eukaryotes. This implies that these subfamilies of proteins are of ancient origin. The evolutionary studies revealed that Ago proteins have undergone a high degree of gene duplication, especially in plants and metazoans, followed by diversification in their function. The numbers of Argonaute genes range from 1 (only 1 AGO) in *S. pombe, *8 in mammals (4 AGO, 4 Piwi) to 27 in *C. elegans*. This diversification and specialization of Argonaute has led to the evolution of new classes of small RNAs and regulatory pathways in several lineages. There are also cases of over simplification and loss of Argonaute proteins in different lineages. *S. pombe* has only one AGO protein and plays a role in both post-transcriptional gene silencing and heterochromatin silencing [50]. Whereas, *S. cerevisiae,* another species of the same genus, and a few parasitic organisms such as *T. cruzi* and *L. major,* do not encode Argonaute proteins. It is believed that these organisms might have lost their Argonaute genes as well as the entire RNAi machinery in due course of evolution [51].

AGO subfamily proteins are ubiquitously expressed in many organisms and their number varies from species to species. A human has 4 genes, but the *Arabidopsis *plant has 10 genes. Three of the Ago protein genes in humans, Ago1, Ago3 and Ago4 are located in cluster on chromosome 1, whereas Ago2 gene is located independently on chromosome 8. Among these 4 proteins, Ago2 is the only member that possesses slicer or endonucleolytic cleavage activity and performs miRNA biogenesis and function. The slicer activity is not yet reported in the other 3 proteins, even though the catalytic residues of Ago2 are also conserved in these members [52]. Among the 10 Ago proteins in *Arabidopsis thaliana*, only three of them, Ago1, Ago4 and Ago7 have slicer activity. It is therefore still unclear what the potential roles played by other Ago proteins in these organisms. Why is human Ago2 different in function from its group members, in spite of the conservation of catalytic residues among them? Does it have any evolutionary implication? Being located as clusters, do Ago1, Ago3 and Ago4 have identical functions, but quite different from those of Ago2? These questions can be explained by further research on Ago proteins, especially through structural biology studies of these proteins bound to small RNAs. These studies will also lend an insight into the evolutionary genomics and selection pressure of Ago proteins. 

## EVOLUTION OF miRNAs

We will discuss some of the important mechanisms pertaining to the evolution of miRNAs and their targets. 

### Seed Shifting

**Fig. (3) Notable models showing the mechanism of miRNA evolution.**
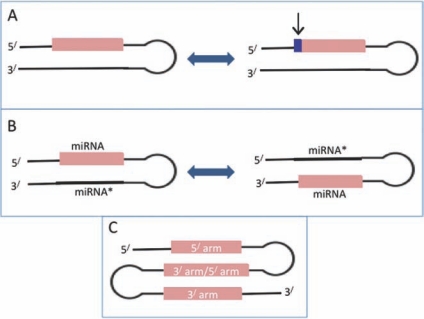
Models depicting (**A**) seed shifting event of miRNA evolution, (**B**) arm switching event of miRNA evolution. In (**A**) the 5^/^ arm encodes the mature miRNA and in (**B**) mature miRNA is encoded from 3^/^ arm of the hairpin precursor miRNA. (****C) Hairpin shifting model of miRNA evolution.

As reported and widely accepted by many researchers, seed, i.e. nucleotide positions 2 through 7 or 8 of 5´ end of the mature miRNA, is considered the most dominant functional unit responsible for exerting its regulatory effect on target mRNAs by binding to 3´ UTR (or CDS, sometimes 5´ UTR). A single nucleotide change in this region may create a new target profile altogether of miRNA. Although miRNAs are conserved across species, some of them often adopt a new evolutionary process, known as seed shifting to evolve a new function that is evolutionary selected [17, 20, 53]. In this process, the sequence of a mature miRNA is moved one or several nucleotides relative to its original position (refer to Fig.**3A**). Wheeler *et al*. reported cases of evolutionary stable shifts in 18 animal species using 454 sequencing of small RNA libraries coupled with genomic searches [53]. The evolutionary stable shifts refer to the movement of 1-2 nt of a mature miRNA sequence to either 3´ or 5´ end of the miRNA and are conserved between two or more taxa. Wheeler *et al*. observed two different types of seed shifts; (a) a shift in the nucleotide position 1 with respect to other taxa, due to addition of a nt at 5´ end, (b) a shift in the seed sequence of a paralogous gene that differs in position 1 with respect to the other copies of a gene, both in same taxon as well as in the other taxa. Recently, it is reported that ~13% of conserved miRNAs between *Drosophila* and *Tribolium* exhibit seed shifting [54]. Seed shifting often alters the predicted target profile and thus imparts new or modified functions to the miRNA. The diversity of miRNAs observed within a species primarily results from the duplication of miRNA genes to generate new paralogs. This duplication event, if followed by a seed shift, will help primitive miRNA to acquire new function by altering the predicted targets.

### miRNA* Species

miRNA cloning, sequencing and high throughput studies have now strengthened the idea of the possibility of two mature miRNAs being generated from a single pre-miRNA. Based on the relative abundance of two mature miRNAs, the predominantly expressed miRNA is assigned regular nomenclature, whereas miRNAs from the opposite arm are named as miRNA* [55]. These miRNA* species were initially considered physiologically irrelevant, however, recent studies have shown that miRNA* species can regulate expression of the target genes by loading into RISC efficiently [56]. Recent studies in *Drosophila* have shown that sequences from opposite arms of a pre-miRNA can be incorporated into different Ago complexes. The predominant arm gets into Ago1 and directs translational repression, whereas the miRNA* species direct translational degradation through Ago2 [57, 58]. This functional acquisition by the miRNA* species since geologic times is considered as an evolutionary event and demonstrates commendable and complex impact on gene regulatory networks as reported in vertebrates [59].

### Arm Switching

miRNA families sometimes undergo a transition from miRNA* species to predominant mature miRNA and vice Models depicting (**A**) seed shifting event of miRNA evolution, (**B**) arm switching event of miRNA evolution. In (**A**) the 5^/^ arm encodes the mature miRNA and in (**B**) mature miRNA is encoded from 3^/^ arm of the hairpin precursor miRNA. (**C**) Hairpin shifting model of miRNA evolution.

versa through altered arm usage (Fig.**3B**). This phenomenon, known as arm switching [54] is observed in different tissues and developmental times [17, 60]. As an example of arm switching, the miR-100/10 family that consists of 3 conserved subfamilies, miR-125, miR-10 and miR-993 were generated by the duplication of miR-100, the only miRNA shared by all bilateria [61]. Most of the mature miRNAs from the miR-100/10 family originate from the 5´ arm of the pre-miRNAs, however, the predominant miRNA from the *Drosophila* miR-10 locus originates from the 3´ arm [62] as in the case of miR-993 in *D. melanogaster *[63], *L. migratoria *[64], and *P. clarkii* [53]. miR-100 and miR-125 show arm switches in different tissues of mammals [65]. The arm switching in miRNA families of chicken is observed in developmental time course [66]. This event of change in arm usage is considered as a functional evolution of the miRNAs, however, the molecular mechanism underlying this change is unknown. The choice of arm during Dicer cleavage is presumed to depend on thermodynamic properties of the duplex [67]. Despite the identical thermodynamic properties of miR-10 sequences in *D. melanogaster* and *T. castaneum*, they display opposite arm usage [61]. This suggests that the thermodynamic feature alone may not explain the reason for the choice of arm. The determinants (structure or sequence signals) for the choice of arm may be located outside the duplex, probably in the loop and/or flanking regions. Marco *et al.* reported that ~11% of the orthologous miRNA pairs in *Drosophila* and *tribolium* show arm-switching events [54]. The arm switching is likely to have a commendable impact on the evolution of target gene network and may lead to the evolution of function of a miRNA. The duplication of an ancestral pre-miRNA that encode mature miRNAs from both arms, followed by regulated arm choice among paralogs, leads to sub-functionalization [61]. Whereas duplication of a miRNA that encodes a single mature miRNA, followed by arm switching leads to neo-functionalization [63], imparting new functions to the miRNA by changing the target profile. Aside from arm switching, hairpin shifting is another interesting evolutionary event that leads to the loss of one arm and birth of a new arm in the pre-miRNA, thereby generating a new mature miRNA from the same precursor (refer to Fig.**3C**). This event is observed in some miRNAs of worms [17]. 

### Post Transcriptional Editing of miRNAs

**Fig. (4) RNA editing of a miRNA seed.**
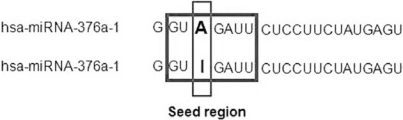
Example of a miRNA that is edited in human. The A-I conversion at position 4 is significant as it redirects targeting capacity.

RNA editing is a different strategy to change a miRNA sequence, and thus diversify its function. In this process, adenosine (A) residues are converted into inosines (I) by the selective action of an adenosine deaminase within dsRNA [68]. Since animal miRNA precursors are necessarily composed of dsRNA, it is conceivable that some miRNAs are edited so as to affect their processing and/or their target set. miR-376 serves as a good example to demonstrate this event (Fig.**4**). A highly edited site is positioned in the middle of its “seed” region. Hence, the genomic and edited versions of miR-376a-5p are predicted to target entirely different sites. Further more, the work of Kawahara *et al*. verifies this experimentally [69]. Other miRNA genes have been found to be edited at various positions [70]. Editing of pre-mir-151 hairpin blocks its cleavage by Dicer [70] which shows that editing might theoretically affect processing and/or loading of miRNAs, with subsequent effects on target regulation.

### Duplication Events

There are a few note-worthy models that proposed the origin and evolution of miRNAs. A model put forward by Allen *et al*., in 2004, postulated that the recently evolved genes encoding miRNAs in plants are originated by inverted duplication of target gene sequences that formed the arms on each side of their respective foldback precursors [71]. This model reported that MIR161 and MIR163 of *A. thaliana* evolved relatively recently through inverted duplication. This hypothesis was further supported by a subsequent study [72]. Most of the miRNA families of *A. thaliana* and *O. sativa *are conserved, however, orthologs of few miRNA families, MIR157, MIR158, MIR165, MIR405 and MIR447 are not found in *O. sativa*. These non-conserved miRNA families share nearly identical nucleotide sequences throughout the length of pre-miRNAs and conserved regions within the promoter sequences between the family members. This hypothesized that non-conserved miRNAs might have evolved by duplication events after the separation of the monocotelydoneous and dicotelydoneous plants ~150 million years ago. Furthermore, in a recent work, the origin and evolution of miRNAs in the exons of protein-coding genes in animals have been explored [73]. Inverted duplications in protein-coding genes may form a locus with regulatory potential to control gene or gene family of origin. 

The functionality of this locus may be evolutionarily selected if miRNA arising from this location is co-expressed and regulation of its progenitor gene is advantageous. In due course of time, mutation and other spontaneous events around this locus may lead to an imperfect pairing in the foldback structure and adaptation to the specialized miRNA-biogenesis machinery. In due course of evolution, miRNAs are also known to originate from processed pseudogenes [74]. Another group of miRNAs in animals originates from genomic repeats and transposable elements [75-78]. C19MC, the largest human miRNA gene cluster ~100-kb long located on chromosome 19, consists of 46 tandemly repeated, primate-specific pre-miRNA genes that are flanked by Alu elements and embedded within a ~400- to 700-nt long repeated unit and rely on non-protein-coding RNA polymerase II for transcription [79]. These findings ascertain the possibility that repeat elements contribute partially to the origin and evolution of miRNA genes. miRNA families are also originated by the duplication of individual genes and clusters. Some miRNA family genes even witnessed a complex expansion by segmental duplications as well as *Alu*-mediated recombination events [80]. The members of miRNA families appear redundant, but might function additively to enhance the regulatory effect on targets or regulate specific targets by their differential expression patterns in different tissues. 

## EVOLUTION OF miRNA TARGETS

miRNAs post-transcriptionally down-regulate the gene expression by binding target mRNAs. Analysis of the evolution of miRNA binding sites is helpful in understanding the co-evolution between miRNAs and their targets. The functionally critical and spatially or temporally expressed ‘non-housekeeping’ genes are stringently regulated by miRNAs through their longer 3´UTRs that harbor more target sites for regulation. However, the ubiquitously expressed ‘housekeeping’ genes tend to have shorter 3´UTRs to avoid miRNA targeting [81]. Bioinformatic studies suggested that human genes that are co-expressed with miRNAs avoid evolving the target seed for those miRNAs [81-83]. Even, the target sites are lost/depleted in orthologs from related species, which suggests that these genes are under pressure to avoid regulation by miRNAs. 

In plants, Guo *et al*. have studied the evolution of miRNA binding sites. A comparative analysis of miRNA-targeted duplicated gene pairs derived from a whole genome duplication (WGD) event was carried out by them in combination with a population genetics study of six experimentally validated miRNA binding sites in rice (*O. sativa*) [84]. Mutations in miRNA target sequences are also observed [85]. These mutations are often found to be functionally significant and might have undergone positive selection for the deletion of targets, thereby acting a rapid way for evolutionary fine-tuning of gene expression. 

## CONCLUSIONS

miRNA research has profoundly changed the perceptions of the role of RNAs from rather uninteresting carriers of coding information to key players in cellular regulation. Indeed, these micro-regulators affect the gene expression at multiple levels. The evolution of miRNA-mediated RNA silencing as a regulatory mechanism in eukaryotes has involved proliferation and specialization of all the core components of RNA silencing pathways. Dicer proteins and processing co-factors have specialized to produce unique miRNAs for various downstream processes. Argonaute proteins have proliferated and evolved a range of functions for amplification and biogenesis of mature miRNAs, enzymatic cleavage of RNA targets, translational repression and recruitment of chromatin-modification factors. Numerous specialized miRNA genes have emerged and evolved as post-transcriptional regulators of gene expression. Furthermore, seed shifting and arm switching events have altered target specificity. The products of these specialization events now constitute an array of diverse small-RNA-based regulatory pathways that control gene expression. Importantly, we have only begun to explore the function of miRNAs in controlling transcriptional networks. The consequences of this novel picture of eukaryotic regulation need to be explored in more detail, using systems biology approaches. Studying the evolutionary history of miRNA genes and their targets reveals an RNA-based gene regulatory layer, implying an additional source for genome plasticity. Further such studies lead us to certain burning questions regarding how these regulatory switches contribute to an increase in genome complexity and how they lead to the emergence of novel traits. These need to be explored further. Retracing the steps along the path of evolution of ncRNAs is a promising approach towards revealing the relationship between different species. The miRNA processing machinery clearly shares main features, and at the same time possesses enough flexibility to allow the acquisition of novel RNA substrates. Overall, it is important to overcome the challenges that lie in identifying the functional roles of miRNA-based regulation in specific tissues and at the whole-organism level that will be crucial to a more integrative view of how miRNA-mediated gene silencing functions in a cellular and developmental context. 

## Figures and Tables

**Fig. (2):**
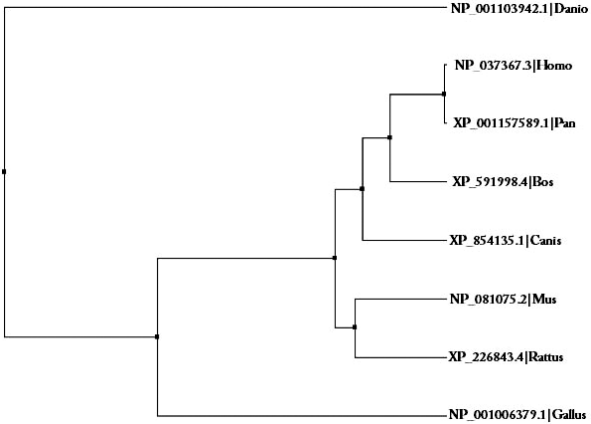
Phylogenetic tree of Drosha proteins in vertebrates. Each node is represented by protein id and genus name.
